# Supplementary material for: Unveiling the Molecular Mechanisms of Glioblastoma through an Integrated Network-Based Approach
Source: Biomedicines. 2024 Oct 1;12(10):2237. doi: 10.3390/biomedicines12102237 (PMC11504402; doi:10.3390/biomedicines12102237)
Supplement: Supplementary file 1 [file biomedicines-12-02237-s001.zip › Supplementary File A.pdf]

Supplementary File A

Unveiling the Molecular Mechanisms of Glioblastoma through an Integrated Network-Based Approach

Ali Kaynar, Atakan Burak Ceyhan, Cheng Zhang, Mathias Uhlén, Hasan Turkez, , Saeed Shoaie and Adil Mardinoglu

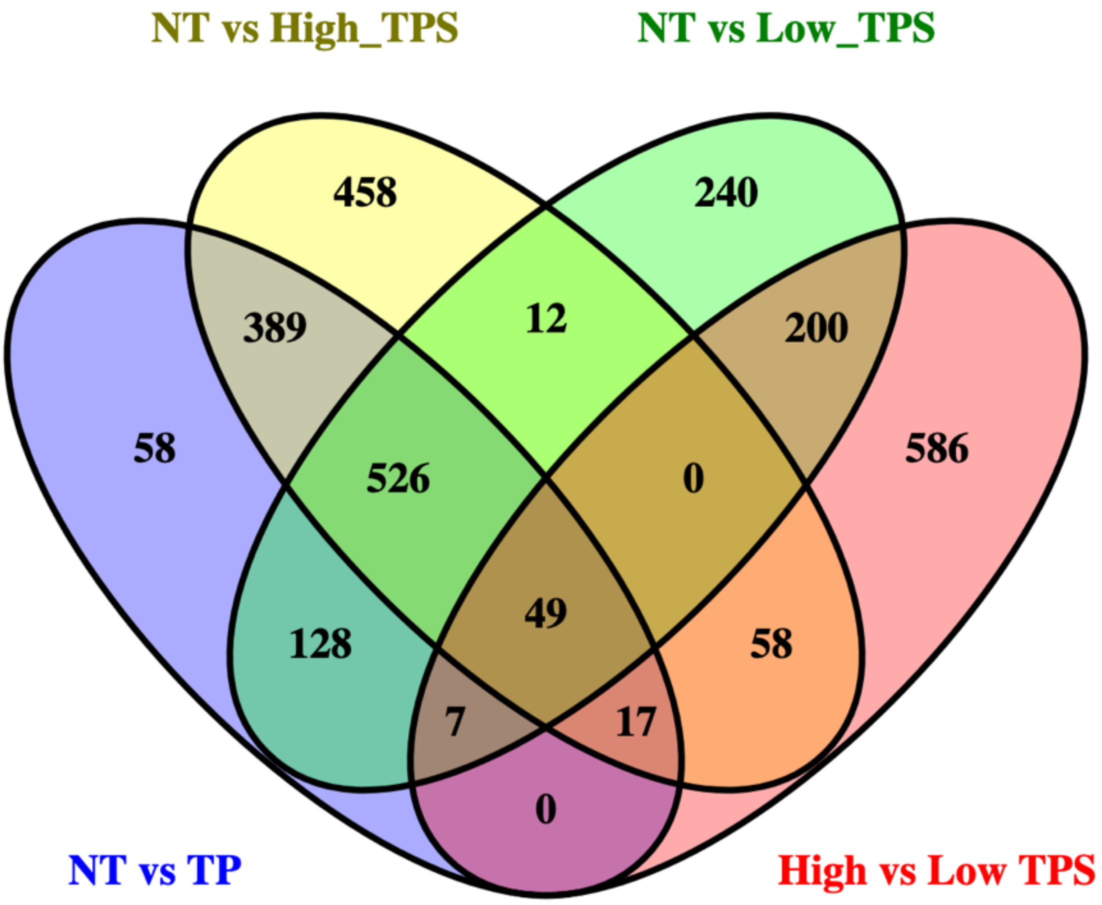

Figure S1. Number of Differential Expression Genes of NT, High TPS, and Low TPS Groups. This figure displays the number of genes significantly altered in glioblastoma sample groups: NT (normal tissue), TP (primary tumour), High TPS, and Low TPS. Genes were identified as significantly altered with an adjusted p-value cutoff of less than 1e−10 and a fold change of at least 1. The analysis highlights the distinct gene expression profiles influenced by tumour proportion.

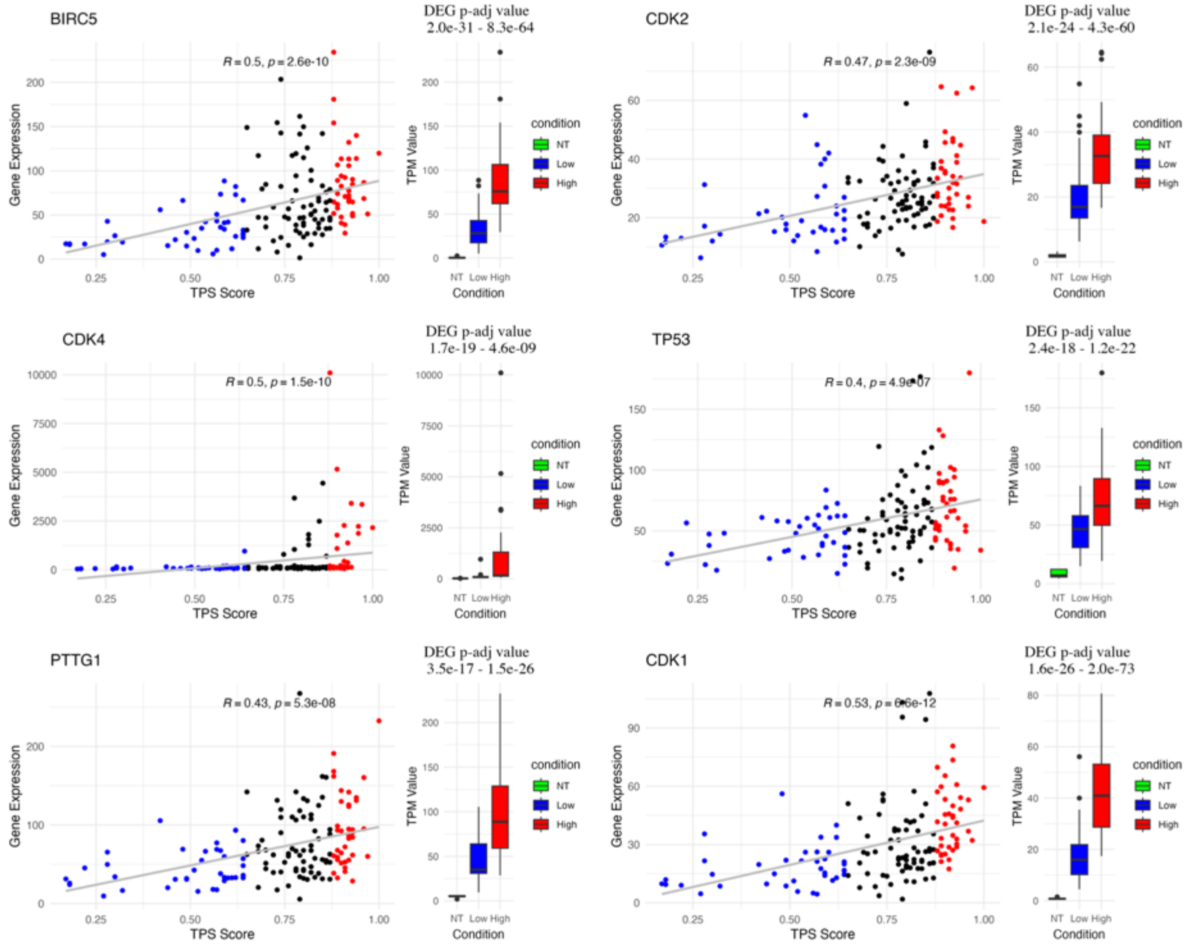

Figure S2. Selected genes from Gene-Set Enrichment Analysis in Glioblastoma Across Different Tumour Purity Scores. This figure illustrates the differential expression of selected genes in glioblastoma categorized by tumour purity: NT (represented in green), High TPS (represented in red), and Low TPS (represented in blue). The p-values shown in the figure caption indicate the level of differential expression, with the left value corresponding to Low TPS and the right value to High TPS.

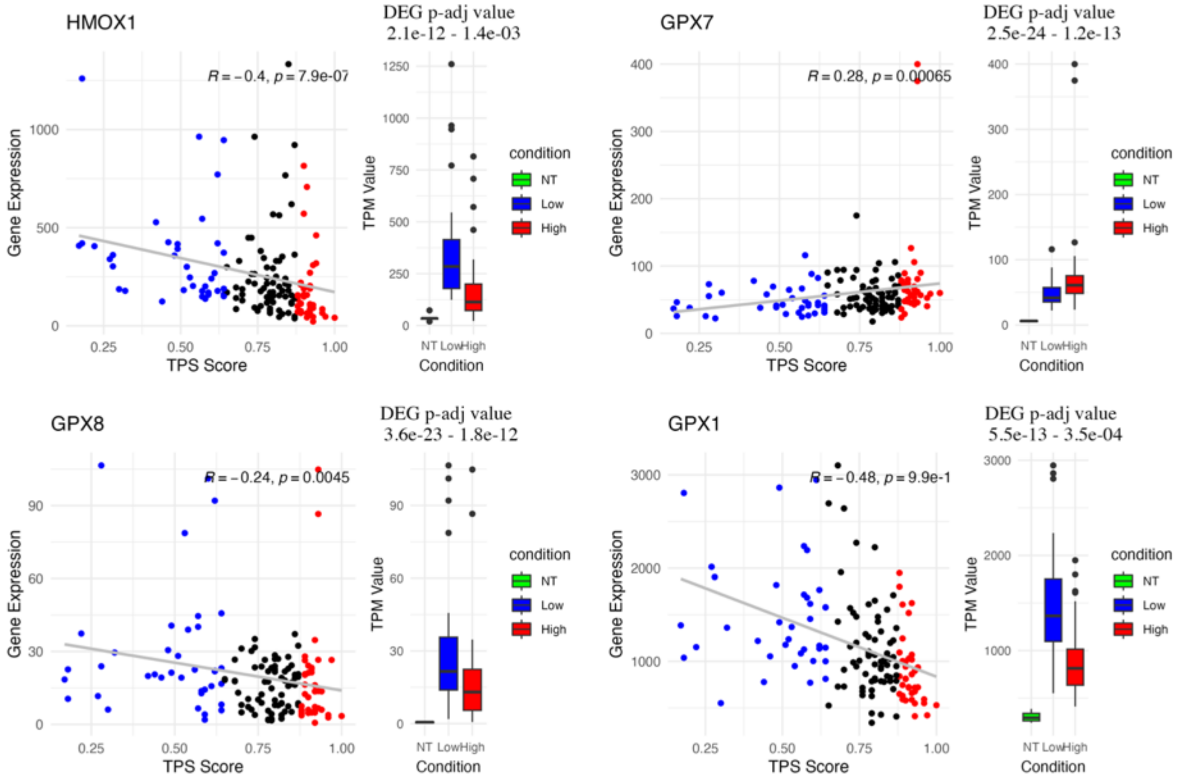

Figure S3. Significant Changes in Heme Metabolism and Oxidative Stress Response Across Metabolic Models. This figure displays the differential expression of key genes associated with heme metabolism and oxidative stress in GBM. NT (represented in green), High TPS (represented in red), and Low TPS (represented in blue). The p-values shown in the figure caption indicate the level of differential expression, with the left value corresponding to Low TPS and the right value to High TPS.

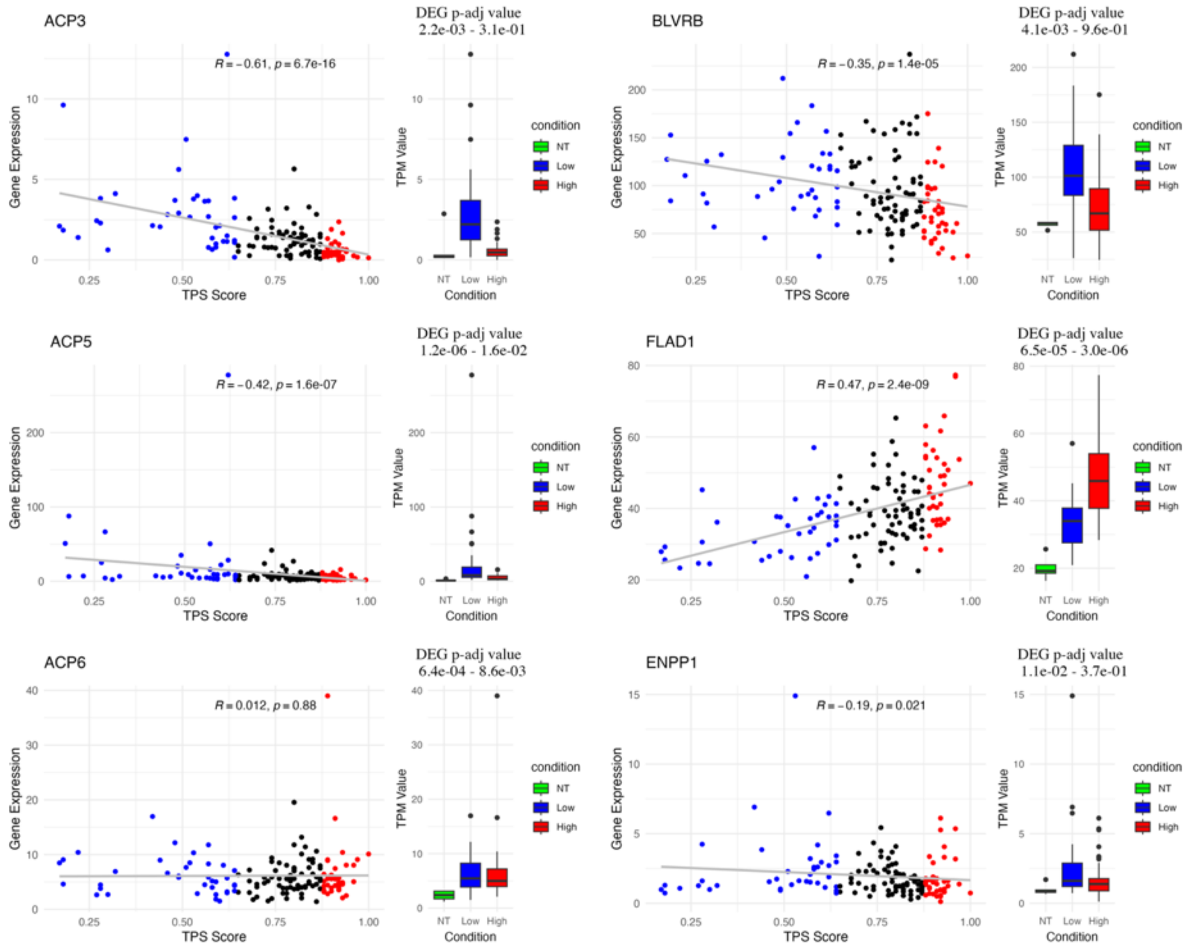

Figure S4. Significant Changes in Vitamin B2 and Riboflavin Metabolism Across Metabolic Models. This figure displays the differential expression of key genes associated with vitamin B2 and riboflavin metabolism in GBM. NT (represented in green), High TPS (represented in red), and Low TPS (represented in blue). The p-values shown in the figure caption indicate the level of differential expression, with the left value corresponding to Low TPS and the right value to High TPS.

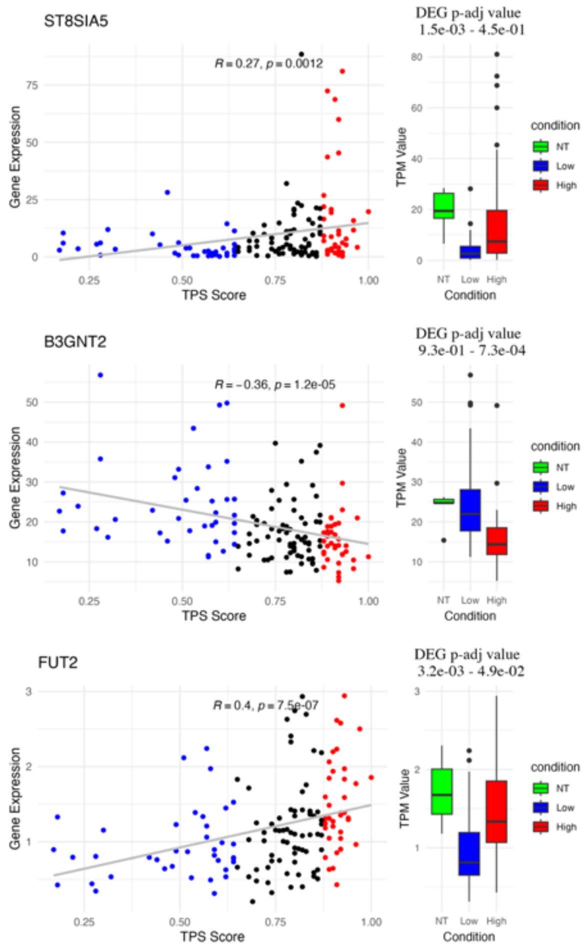

Figure S5. Significant Changes in Blood Group Biosynthesis Across Metabolic Models. This figure displays the differential expression of key genes associated with Blood group biosynthesis, which are involved in antigen representation, in GBM. NT (represented in green), High TPS (represented in red), and Low TPS (represented in blue). The p-values shown in the figure caption indicate the level of differential expression, with the left value corresponding to Low TPS and the right value to High TPS.

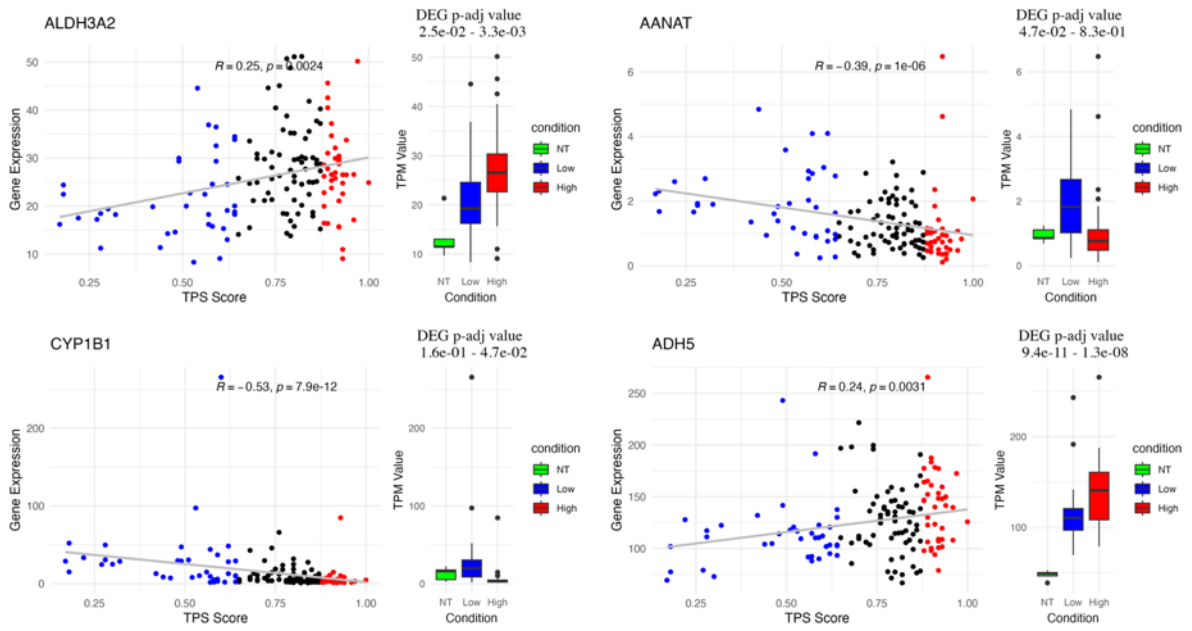

Figure S6: Low TPS Metabolic Models Shows Different Serotonin and Melatonin Biosynthesis Profile. This figure displays the differential expression of key genes associated with Serotonin and Melatonin Biosynthesis in Low TPS model. NT (represented in green), High TPS (represented in red), and Low TPS (represented in blue). The p-values shown in the figure caption indicate the level of differential expression, with the left value corresponding to Low TPS and the right value to High TPS.

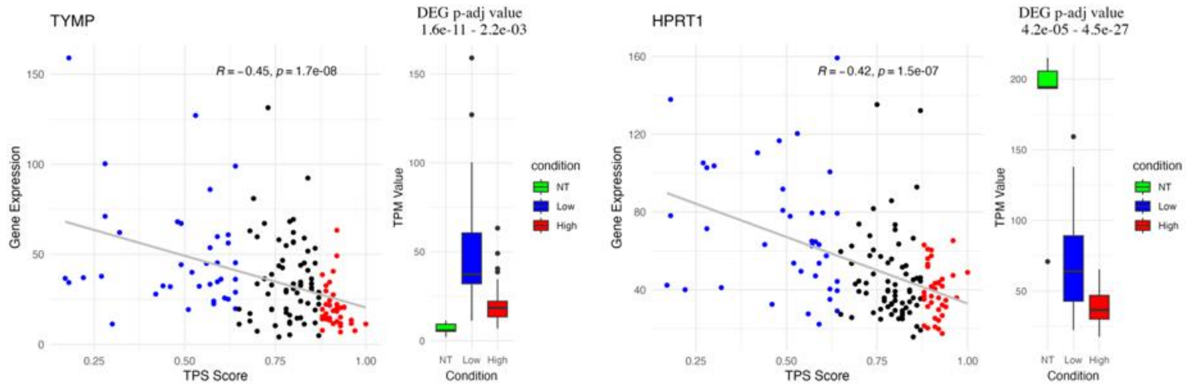

Figure S7: Low TPS Metabolic Model Passed ATP Salvage from Hypoxanthine Metabolic Task. This figure displays the differential expression of key genes associated with ATP salvage from hypoxanthine in Low TPS. NT (represented in green), High TPS (represented in red), and Low TPS (represented in blue). The p-values shown in the figure caption indicate the level of differential expression, with the left value corresponding to Low TPS and the right value to High TPS.

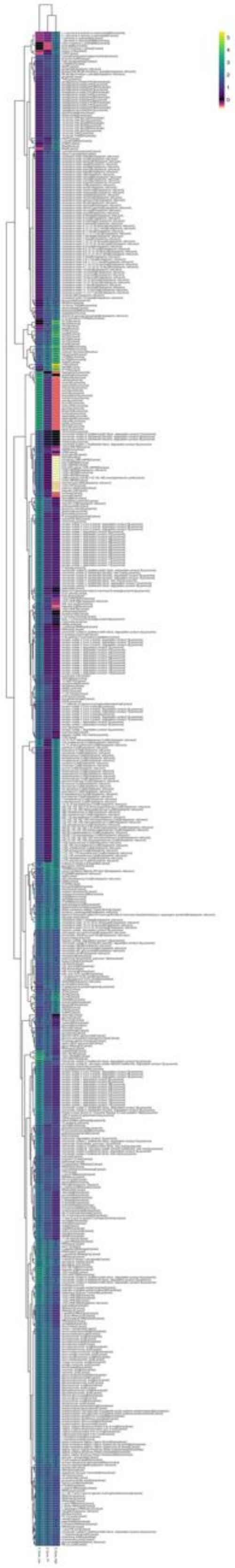

***Figure S8:* URGA Metabolites Across TPS Groups.** The figure illustrates the effects of TPS on GEMs, which indicates URGA (upregulated-gene-affected) reporter metabolites. The colour gradient indicates Z-scores of corresponding metabolites. *, **, and *** indicate significant levels of the p-value as, 0.05, 0.01, and 0.001 respectively.

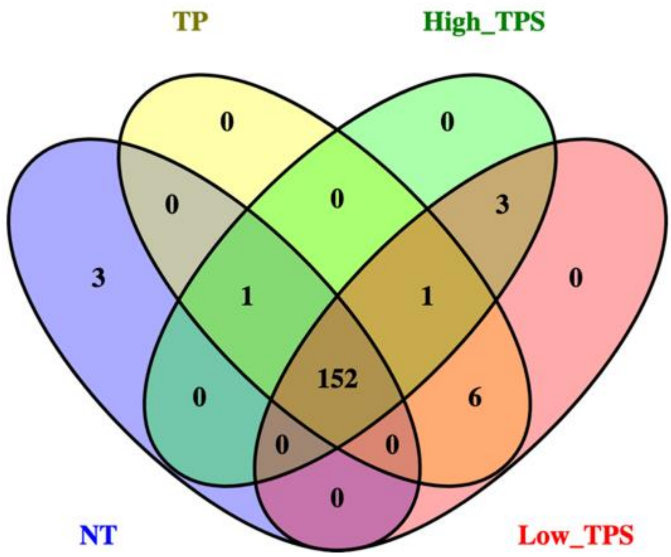

Figure S9. Single Gene Deletion Results for Models. This figure highlights that while the majority of essential genes are shared across different models, there are 10 genes uniquely essential to cancer models, underscoring specific vulnerabilities in cancerous conditions.

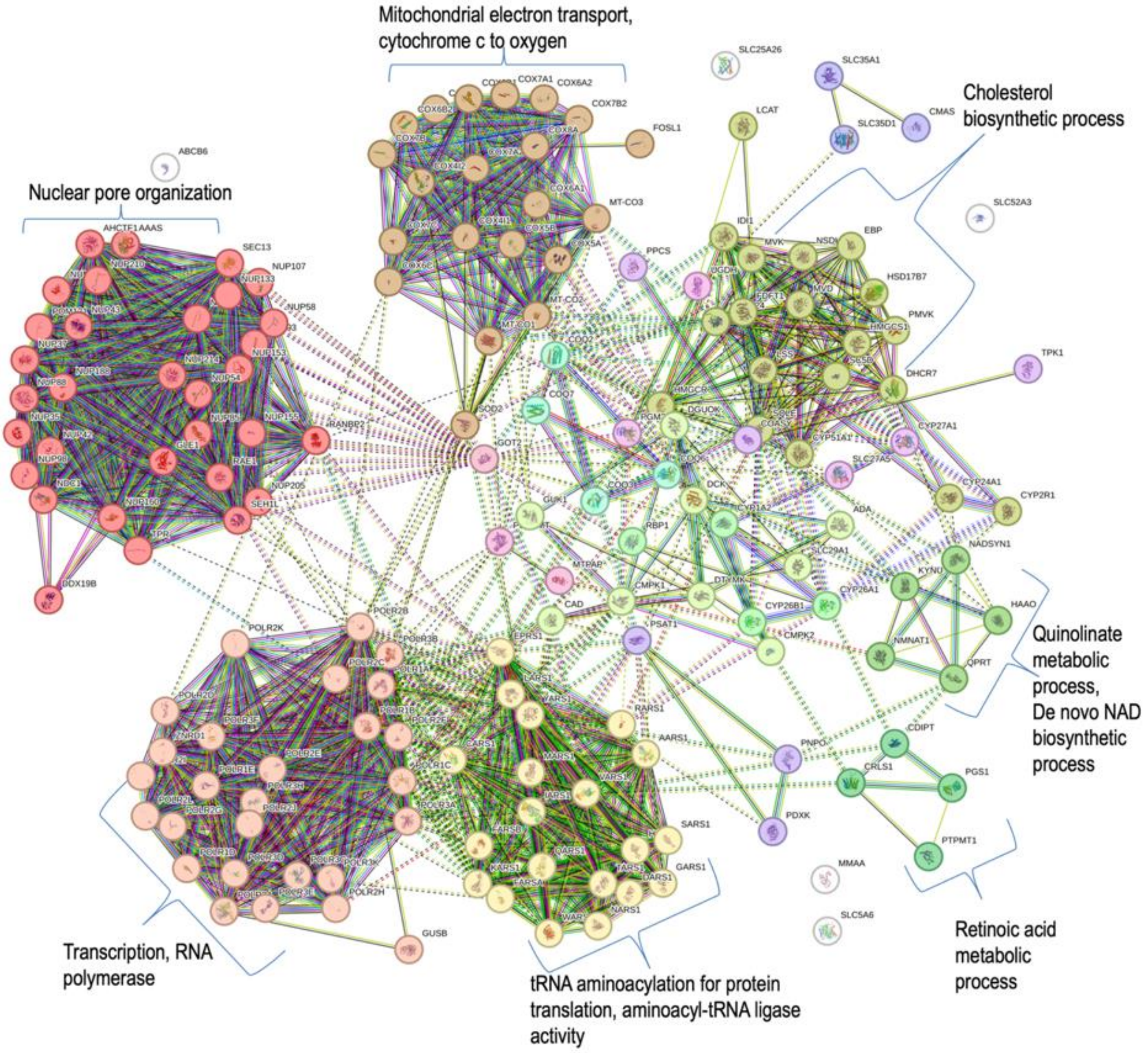

Figure S10: STRING Network Analysis of Essential Genes from Single-Gene Deletion. This figure demonstrates that essential genes predominantly cluster into five major groups, along with various other metabolic processes.

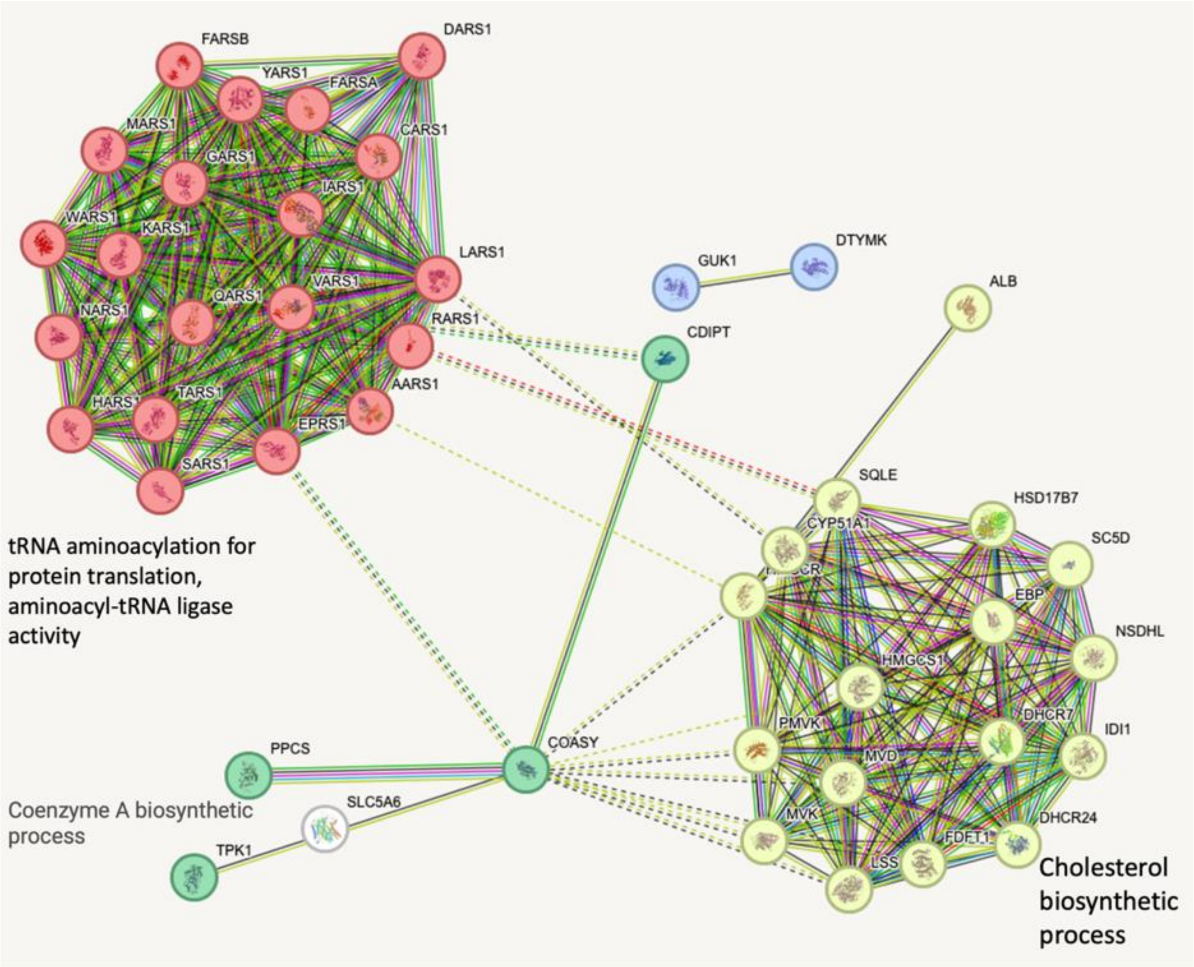

Figure S11: STRING Network Analysis of Essential Genes from Metabolic Task. This figure demonstrates that essential genes predominantly cluster into two major groups, along with one other metabolic process.

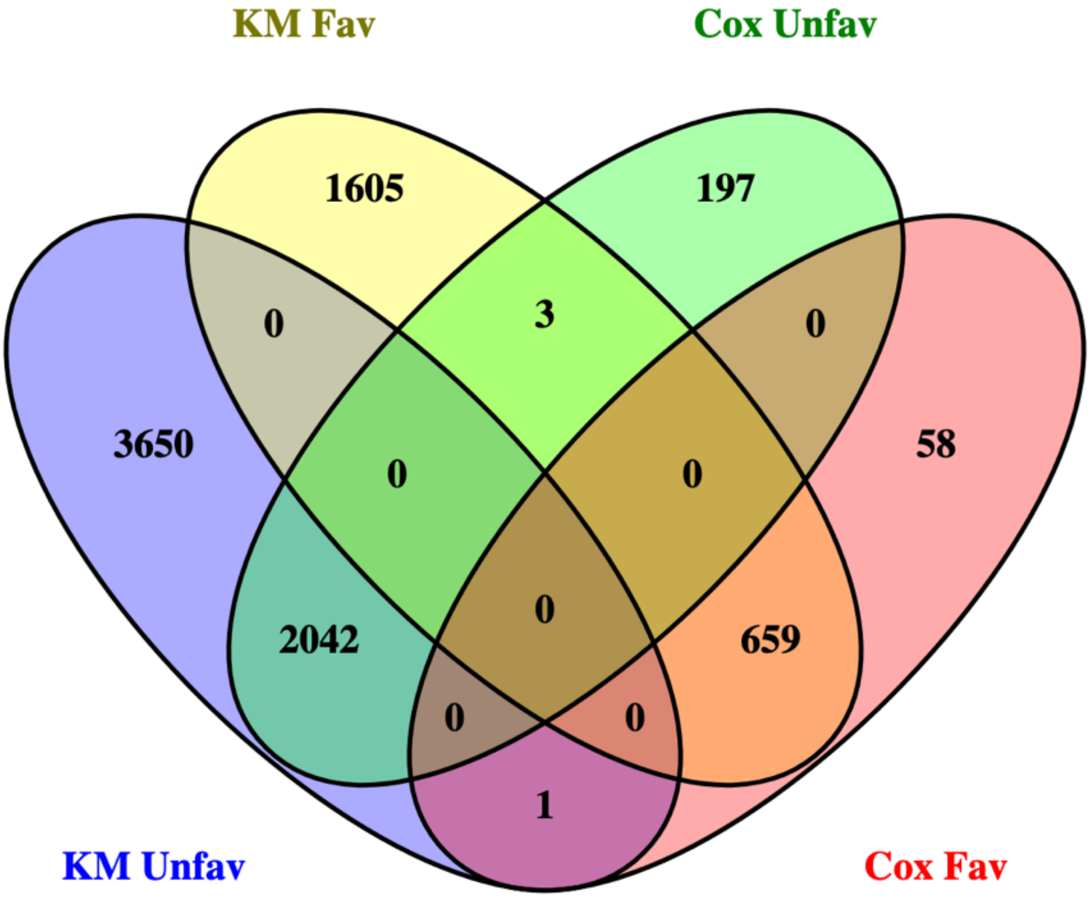

Figure S12: Intersection of Prognostic Gene Sets in Survival Analysis via Kaplan-Meier and Cox methods. This Venn diagram displays the distribution and overlap of prognostic genes identified through Kaplan-Meier and Cox proportional hazards models, categorized into favourable and unfavourable outcomes

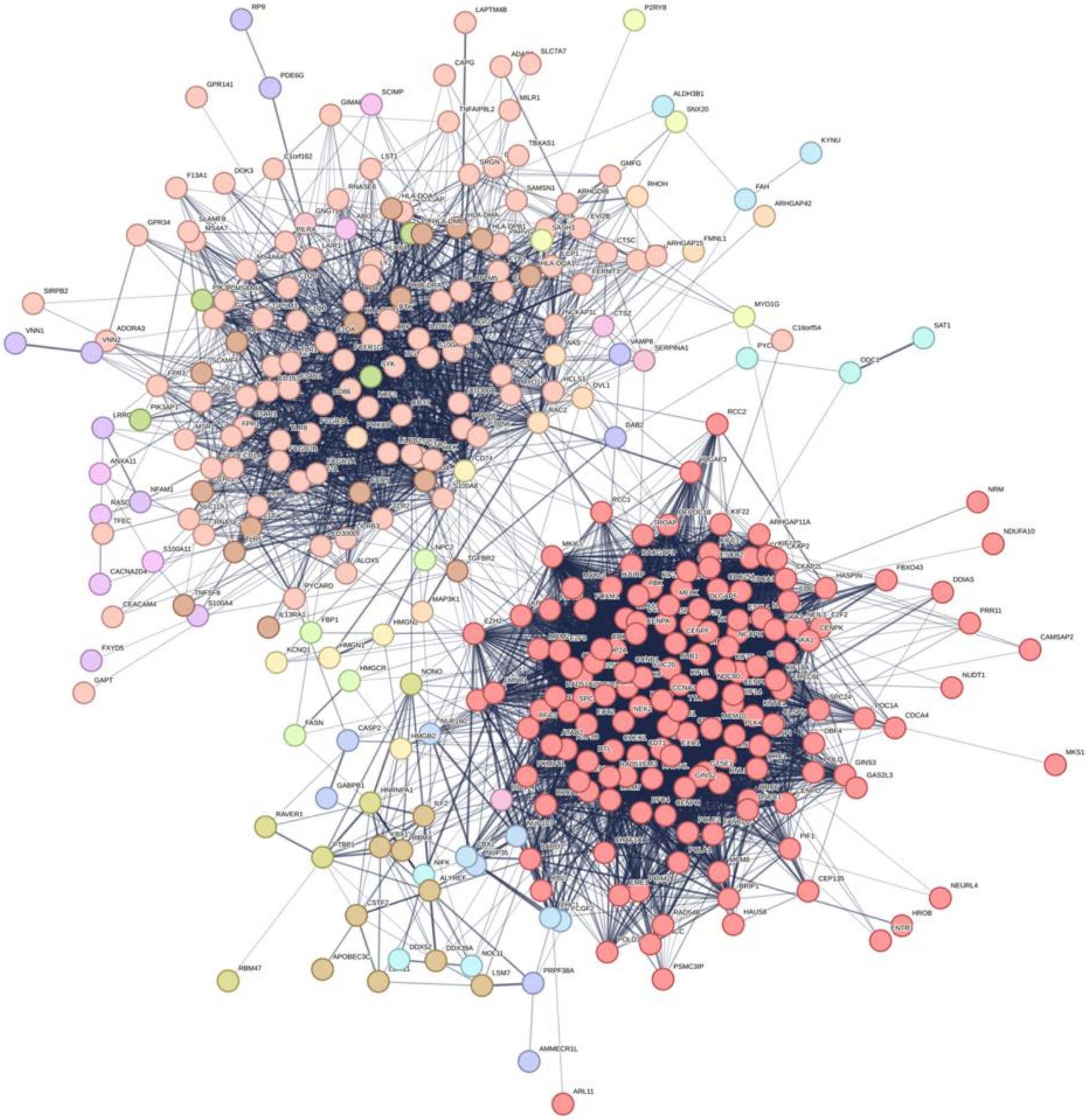

Figure S13: **STRING Network of Correlated and Significant Genes in GBM.** This figure displays a STRING network analysis of 371 genes identified by their high correlation and significant expression changes in GBM. The network segregates these genes into clusters based on their biological roles, emphasizing their involvement in immune processes and cell division. Colour represents clusters (Supplementary File B)

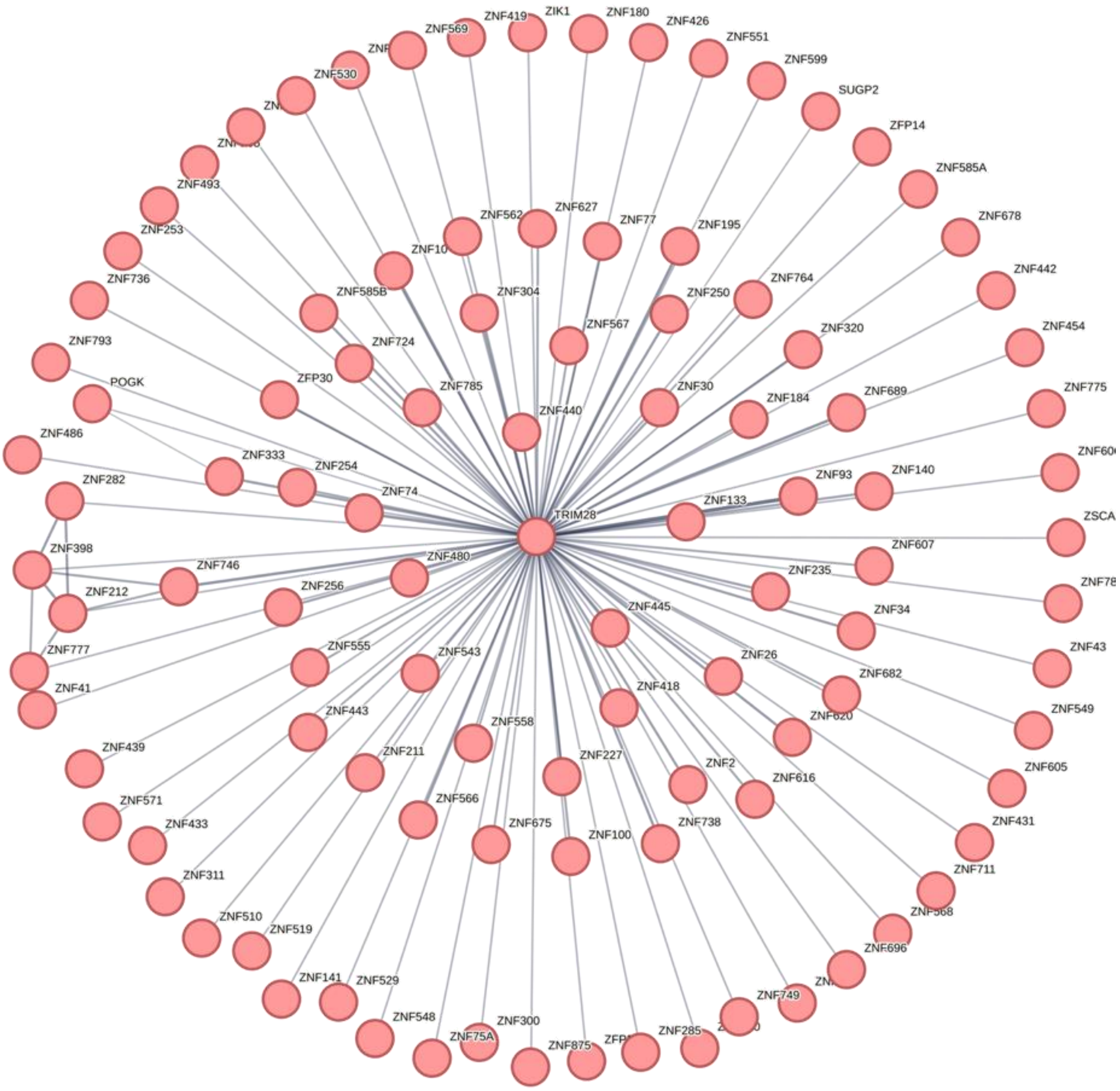

Figure S14: STRING Network of TREM28 and zinc finger family members. This figure displays a subnetwork of positively correlated (R>=0.5) genes with a physical STRING network. The network segregates these genes into clusters based on their biological roles, emphasizing their involvement in the regulation of gene expression.

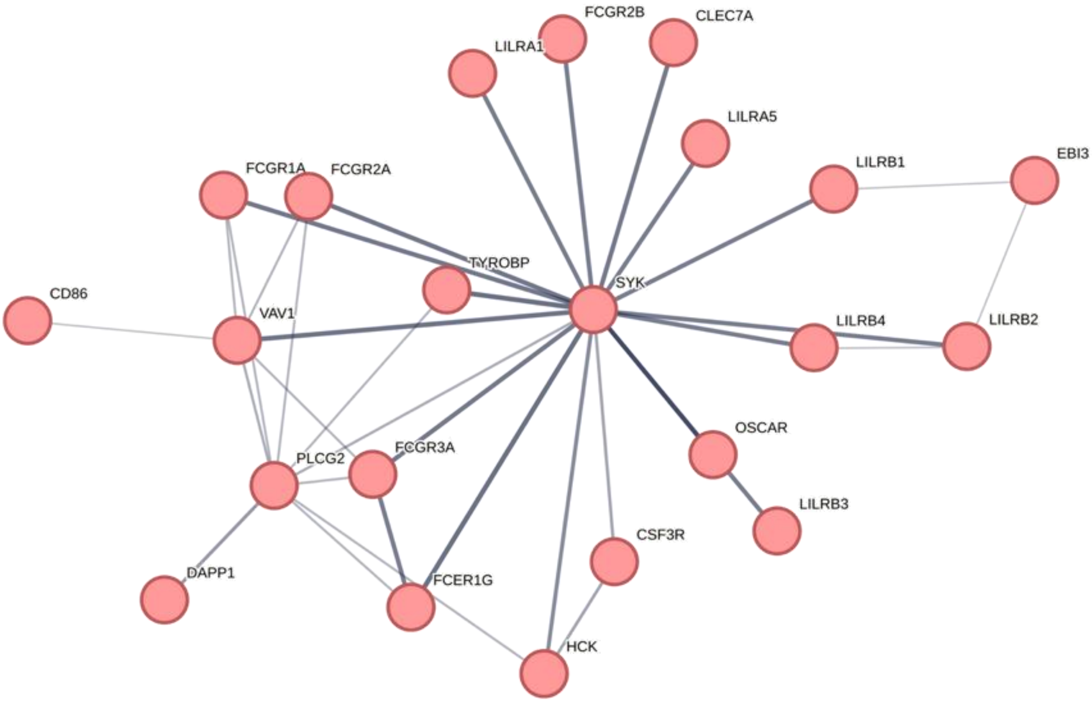

Figure S15: STRING Network of SYK gene. This figure displays a subnetwork of negatively correlated (R<=-0.5) genes with a physical STRING network. The network segregates these genes into clusters based on their biological roles, emphasizing SYK interaction with LILRs involvement including LILRB4 in the regulation of immune responses.
